# Intramuscular Port-Site Hernia after Robot-Assisted Colorectal Surgery despite Fascial Closure: A Case Report

**DOI:** 10.70352/scrj.cr.26-0125

**Published:** 2026-06-13

**Authors:** Shunsaku Furuke, Yusuke Nakamichi, Naoki Yagi, Norihiro Ishii, Shigemasa Suzuki, Takashi Ooki, Ryusuke Aihara, Akira Mogi, Yasuo Hosouchi

**Affiliations:** Department of Surgery, Saiseikai Maebashi Hospital, Maebashi, Gunma, Japan

**Keywords:** port-site hernia, intramuscular hernia, robot-assisted surgery

## Abstract

**INTRODUCTION:**

Port-site hernia (PSH) is a rare but potentially serious complication of minimally invasive surgery. Although adequate fascial closure is recommended, PSH may occur even after apparently appropriate closure. Intramuscular PSH, in which the bowel becomes incarcerated between abdominal wall muscle layers without overt fascial dehiscence, is particularly uncommon.

**CASE PRESENTATION:**

An 80-year-old woman underwent robot-assisted right hemicolectomy and high anterior resection using the da Vinci Xi surgical system (Intuitive Surgical, Sunnyvale, CA, USA). A 12-mm robotic port was placed in the right lower abdomen, and the external oblique aponeurosis at the port site was closed with a single interrupted suture. On POD 8, she developed symptoms of bowel obstruction. CT revealed incarceration of the small intestine within the abdominal wall at the port site. Emergency surgery confirmed intramuscular herniation between the external and internal oblique muscles without fascial dehiscence, and partial small bowel resection was required.

**CONCLUSIONS:**

Intramuscular PSH can occur despite appropriate fascial closure, particularly when a single robotic port is subjected to extensive multidirectional manipulation centered on the remote center. Careful postoperative monitoring and consideration of full-thickness layered closure may help prevent this complication.

## INTRODUCTION

Port-site hernia (PSH) is a recognized complication of minimally invasive surgery, including laparoscopic and robot-assisted procedures. The reported incidence of PSH ranges from approximately 0.5% to 2% in laparoscopic surgery.^1–3)^ PSH after robotic surgery has also been reported, although available data remain limited.^4)^ Most PSHs occur at trocar sites measuring 10–12 mm in diameter and are generally associated with inadequate fascial closure.^2)^ Nevertheless, herniation has been reported even after apparently appropriate closure.^5)^ Intramuscular PSH, in which the bowel becomes incarcerated between abdominal wall muscle layers without overt fascial dehiscence, is particularly rare. We herein report a rare case of intramuscular PSH following robot-assisted colorectal surgery.

## CASE PRESENTATION

**Fig. 1:**
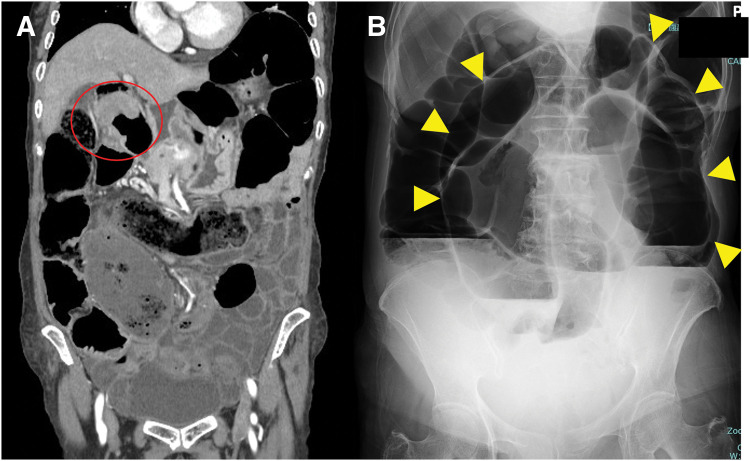
Preoperative imaging findings. (**A**) Contrast-enhanced CT (coronal view) showing a contrast-enhancing wall-thickening lesion located near the hepatic flexure of the transverse colon (red circle). No enlarged lymph nodes or apparent distant metastases were observed. (**B**) Abdominal radiograph demonstrating a markedly elongated sigmoid colon with torsion, consistent with sigmoid volvulus (yellow arrowheads).

**Fig. 2:**
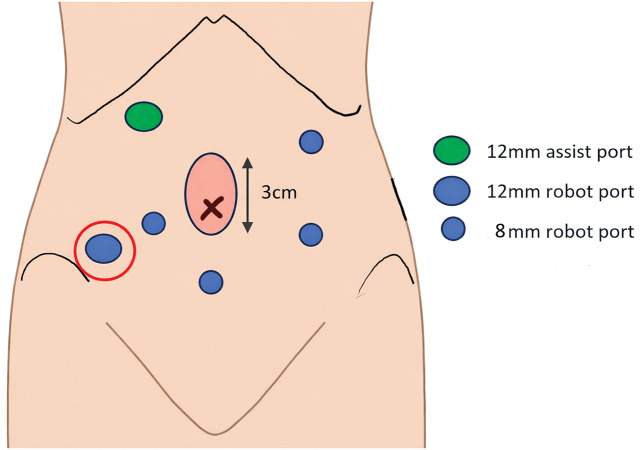
Schematic illustration of port placement during the initial robot-assisted surgery. The right lower 12-mm robotic port (red circle) was the site of hernia incarceration.

An 80-year-old woman with a BMI of 18.7 kg/m^2^ and a medical history of schizophrenia was diagnosed with transverse colon cancer (cT3N0M0, cStage IIA) and sigmoid volvulus. She had no history of smoking and her performance status was 2. Preoperative CT and abdominal radiography revealed a tumor located near the hepatic flexure, as well as a markedly dilated sigmoid colon consistent with volvulus (**Fig. 1**). She underwent robot-assisted right hemicolectomy and high anterior resection during the same procedure in the lithotomy position. The operative time was 259 min, including a console time of 228 min, and the estimated blood loss was 10 mL. Port placement is illustrated in **Fig. 2**. The procedure was performed using the da Vinci Xi surgical system (Intuitive Surgical, Sunnyvale, CA, USA) with a pneumoperitoneum pressure of 10 mmHg. A 12-mm robotic port was placed in the right lower abdomen and was used for both cranial and caudal operative fields, resulting in extensive multidirectional manipulation centered on the robotic remote center. The remote center alignment was confirmed intraoperatively according to the standard protocol. At the end of surgery, the external oblique aponeurosis at the 12-mm port site was closed under direct visualization using a single interrupted 2-0 absorbable suture. This method of port-site closure is our standard practice for 12-mm ports at our institution. Closure of the peritoneum was not confirmed in this case.

**Fig. 3:**
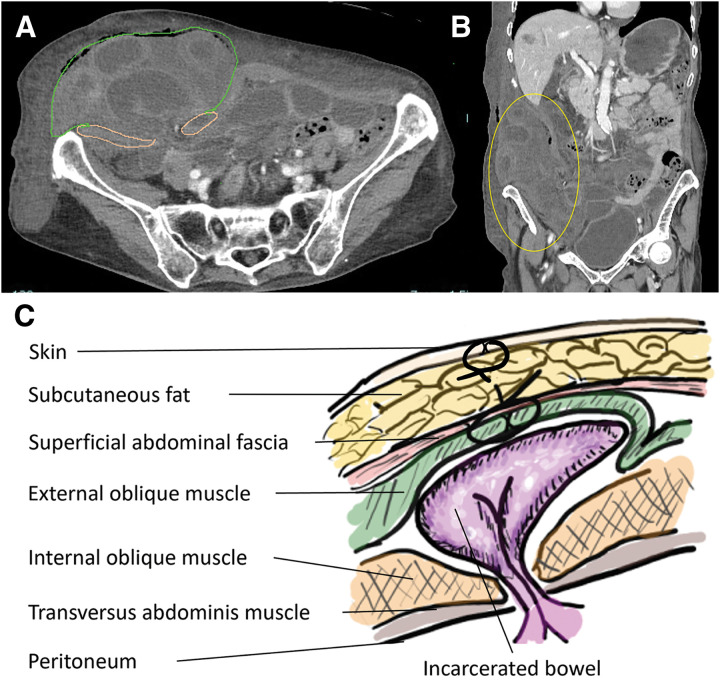
Contrast-enhanced CT and anatomical illustration of the intramuscular port-site hernia. (**A**) Axial CT image showing small bowel incarceration within the abdominal wall. The external oblique muscle (green) and internal oblique muscle (orange) are indicated, demonstrating that the bowel is entrapped between these muscle layers. (**B**) Coronal CT image demonstrating extensive herniation along the right abdominal wall (yellow ellipse). (**C**) Schematic illustration of the abdominal wall layers indicating herniation and bowel incarceration between the external (green) and internal (orange) oblique muscles.

The postoperative course was initially uneventful. However, on POD 8, the patient developed sudden abdominal pain and vomiting. Contrast-enhanced CT revealed protrusion of a long segment of the small intestine into the abdominal wall at the right lower port site. The herniated bowel was located between the external oblique and internal oblique muscle layers, while the peritoneal continuity appeared preserved, suggesting an intramuscular PSH (**Fig. 3**).

**Fig. 4:**
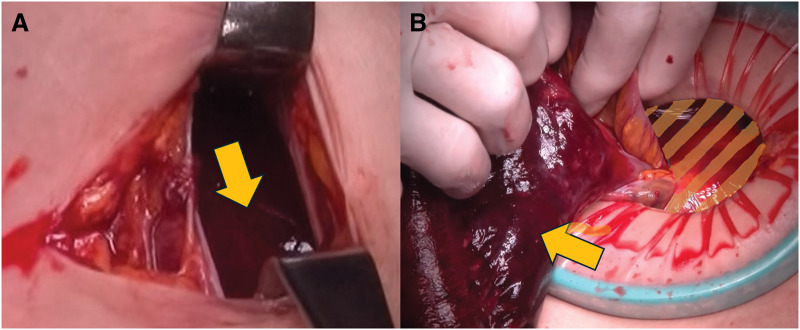
Intraoperative findings of the intramuscular port-site hernia. (**A**) After incision of the external oblique aponeurosis, an incarcerated small bowel was identified within the intramuscular space (arrow). (**B**) The small bowel (arrow) was incarcerated at the hernia orifice between the external and internal oblique muscles. The hatched area indicates the internal oblique muscle, which formed the hernia orifice. The incarcerated bowel showed compromised perfusion, necessitating partial small bowel resection.

Emergency surgery was performed via an anterior approach through reopening of the port-site incision. A skin incision of approximately 6 cm was made. Intraoperatively, the small intestine was found to be incarcerated between the external and internal oblique muscles without obvious fascial dehiscence (**Fig. 4**). The hernia orifice measured approximately 20 mm in diameter. Because ischemic changes were observed, partial small bowel resection of approximately 140 cm was required. After reduction of the herniated bowel, the port site was closed with direct full-thickness suturing using 0 nonabsorbable suture, including all layers of the abdominal wall. The postoperative course after reoperation was uneventful, and the patient was discharged without further complications. No recurrence of PSH was observed during the 4-month follow-up period.

## DISCUSSION

PSH is an infrequent but potentially life-threatening complication of minimally invasive surgery. Reported risk factors for PSH include larger trocar size and inadequate fascial closure, as well as various patient-related and technical factors.^1–3)^ In the present case, repeated multidirectional manipulation of a single robotic port may have contributed to increased mechanical stress on the abdominal wall. In addition, the patient’s low BMI may have further predisposed her to tissue fragility.

Nevertheless, cases of herniation despite apparently adequate closure have been reported.^5)^ Sanada et al. described intramuscular PSH after laparoscopic surgery in which the fascia had been closed appropriately, emphasizing that bowel can become trapped between muscle layers even without fascial dehiscence.^6)^ Our case represents a similar mechanism occurring after robot-assisted surgery.

Several Japanese case reports have described PSH occurring after fascial closure at 12-mm trocar sites.^6–9)^ A review of the domestic literature identified 4 previously reported cases, all of which developed at 12-mm ports and required reoperation (**Table 1**). Among them, 3 cases involved true intramuscular herniation between the abdominal muscle layers. Similar to our case, early incarceration often resulted in bowel resection. These findings indicate that intramuscular PSH after fascial closure is a rare but clinically significant entity that may progress rapidly.

**Table 1:** Previously reported cases of intramuscular port-site hernia after fascial closure at ≥12-mm ports

Author (year)	Country	Surgery	Port (mm/site)	Robotic platform	Fascial closure	Hernia location	Treatment	Outcome
Sugimura (2010)^7)^	Japan	Laparoscopic gastrectomy	12/LLQ	—	Yes	Subfascial	Reduction	Not reported
Kurata (2018)^8)^	Japan	Laparoscopic sigmoidectomy	12/RLQ	—	Yes	EO–IO	Resection	No recurrence (7 months)
Hori (2021)^9)^	Japan	Robot-assisted radical prostatectomy	12/Right flank	da Vinci	Yes	EO–IO	Reduction	No recurrence (7 months)
Sanada (2024)^6)^	Japan	Laparoscopic high anterior resection	12/RLQ	—	Yes	EO–IO	Resection	No recurrence (4 months)
Present case	Japan	Robot-assisted right hemicolectomy and high anterior resection	12/RLQ	da Vinci Xi	Yes	EO–IO	Resection	No recurrence (4 months)

da Vinci Xi, Intuitive Surgical, Sunnyvale, CA, USA; EO–IO, between the external oblique and internal oblique muscle layers; LLQ, left lower quadrant; RLQ, right lower quadrant

Previous studies have suggested that PSH after robotic surgery remains uncommon but clinically relevant. In a large cohort study, Damani et al. found that acute PSHs occurred in approximately 0.1%–0.7% of robotic cases, with several requiring small bowel resection.^4)^ In addition, Swank et al. emphasized the importance of meticulous closure of ports ≥10 mm to prevent postoperative herniation.^10)^ These findings support the importance of both port size and mechanical factors in the development of PSH.

When a single robotic port is used for procedures involving different operative fields, the port may be subjected to repeated cranial and caudal movements. In the present case, combined right hemicolectomy and high anterior resection required extensive multidirectional manipulation through the same 12-mm robotic port. We speculate that this repeated mechanical stress on the abdominal wall contributed to intramuscular disruption and subsequent herniation.

This case highlights that fascial closure alone may not always be sufficient to prevent PSH. Layered closure, including the peritoneum and internal oblique fascia, may be considered in selected situations, particularly when excessive port manipulation or multidirectional stress is anticipated. Surgeons should maintain a high index of suspicion for this complication when early postoperative abdominal symptoms develop.

## CONCLUSIONS

Intramuscular PSH is a rare but clinically important complication that can occur even after appropriate fascial closure in robot-assisted surgery. Awareness of this entity and consideration of full-thickness layered closure in selected situations may help reduce the risk of severe incarceration.

## DECLARATIONS

### Availability of data and materials

The datasets supporting the conclusions of this article are included within the article.
